# Supplementary material for: Ice-marginal proglacial lakes enhance outlet glacier velocities across Greenland
Source: Commun Earth Environ. 2026 Apr 1;7(1):287. doi: 10.1038/s43247-026-03363-9 (PMC13043277; doi:10.1038/s43247-026-03363-9)
Supplement: Supplementary file 2 — Supplementary Information [file 43247_2026_3363_MOESM2_ESM.pdf]

Communications Earth and Environment

Supporting Information for

Ice-marginal proglacial lakes enhance outlet glacier velocities across Greenland

Connie M. Harpur[1], Mark W. Smith[1], Jonathan L. Carrivick[1], Duncan J. Quincey[1], Liam Taylor[1]

[1]School of Geography, University of Leeds, Leeds, United Kingdom

File contents

Supplementary Note S1
Figures S1 to S7
Tables S1 to S3
References

Supplementary Note S1. Glacier selection

Since lake-terminating outlet glaciers of the Greenland Ice Sheet are largely unmapped and missing from glacier inventories (e.g. the Randolph Glacier Inventory (RGI Consortium, 2023) or Global Land Ice Measurements from Space (GLIMS) Glacier Database (GLIMS and NSIDC, 2005)), we systematically chose sample glaciers which fulfil the criteria described in section 4.1. Specifically, outlet glaciers were identified as distinct fields of pronounced, relatively fast flow at the ice sheet margin. These flow fields were assessed against satellite imagery for convergent surface features typical of glacierised flow (Ely et al., 2017).

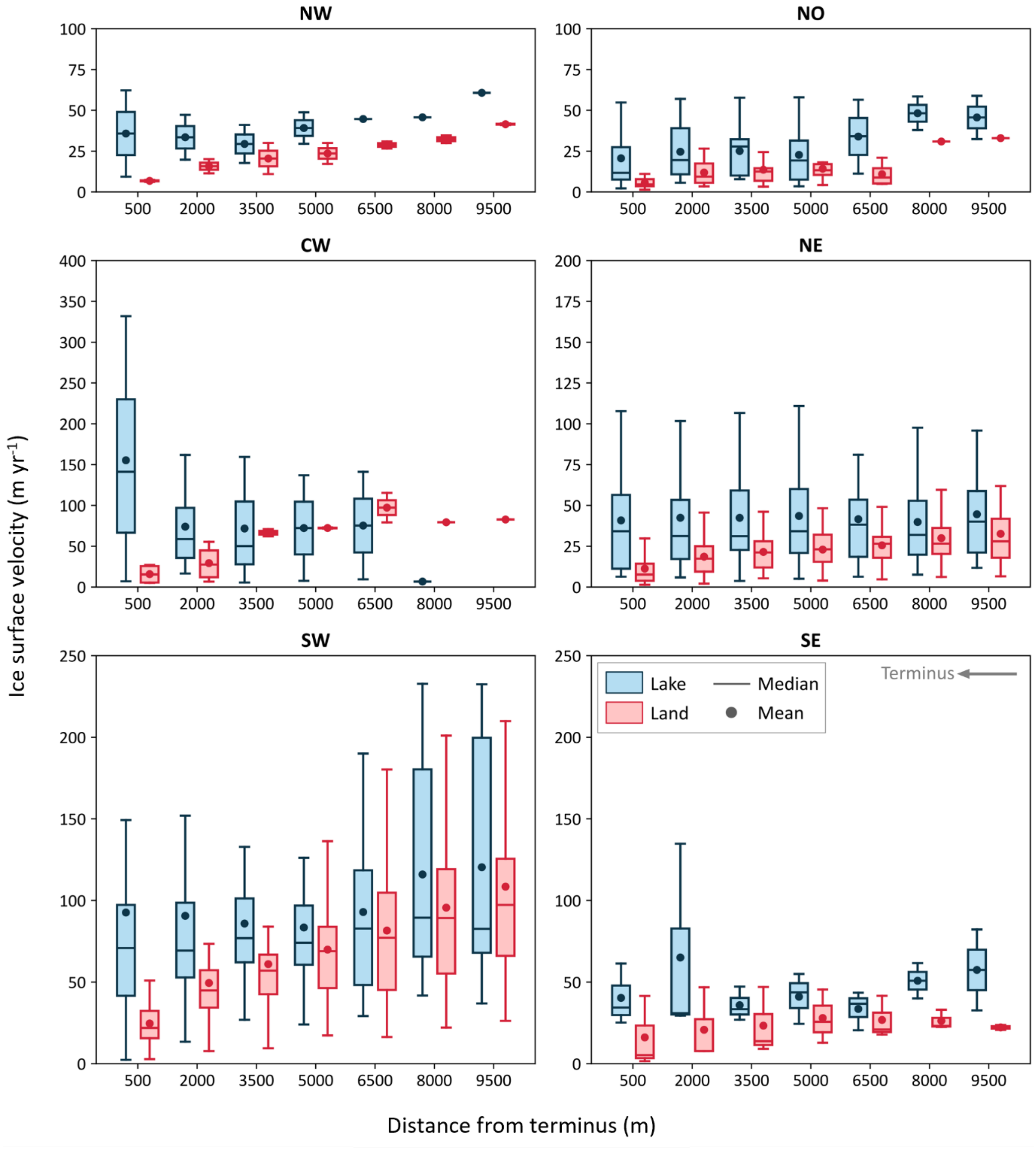

Supplementary Figure S1: Median box-sampled velocity at each flowline location, aggregated from all lake-terminating (blue) and land-terminating (red) glaciers within each ice sheet region. The spread of velocity values across the sample population is shown by the box height. Sample numbers are displayed in Table S1.

Region	Distance from terminus (m)	Lake sample number	Land sample number	U-statistic	*p*-value
**SW**	500	49	49	2032.5	**0.00**
	2000	47	49	1712	**0.00**
	3500	37	47	1255.5	**0.00**
	5000	26	43	669	0.18
	6500	20	35	388	0.51
	8000	13	32	240	0.43
	9500	12	24	162	0.56
**CW**	500	4	4	14	0.11
	2000	4	4	13	0.20
	3500	3	2	2	0.80
	5000	2	2	2	1.00
	6500	2	2	2	1.00
	8000	1	1	0	1.00
	9500	0	1		
**NW**	500	2	2	4	0.33
	2000	2	2	3	0.67
	3500	2	2	3	0.67
	5000	2	2	3	0.67
	6500	1	2	2	0.67
	8000	1	2	2	0.67
	9500	1	2	2	0.67
**NO**	500	15	15	184.5	**0.00**
	2000	14	15	157	**0.02**
	3500	10	11	78	0.11
	5000	7	9	37	0.61
	6500	3	4	10	0.23
	8000	2	1	2	0.67
	9500	2	1	1	1.00
**NE**	500	29	29	701	**0.00**
	2000	27	29	580	**0.00**
	3500	22	29	458	**0.01**
	5000	20	28	399	**0.01**
	6500	17	22	255	0.06
	8000	15	17	153	0.35
	9500	11	16	113	0.23
**SE**	500	3	3	7	0.40
	2000	3	3	7	0.40
	3500	3	3	7	0.40
	5000	3	3	6	0.70
	6500	3	3	6	0.70
	8000	2	3	6	0.20
	9500	2	2	4	0.33

Supplementary Table S1. Statistical comparison of differences between the median velocity of lake-terminating and land-terminating glaciers at each flowline distance within each ice sheet region, using a Mann-Whitney U test for difference and a significance threshold of $p \leq 0.05$ (highlighted in bold).

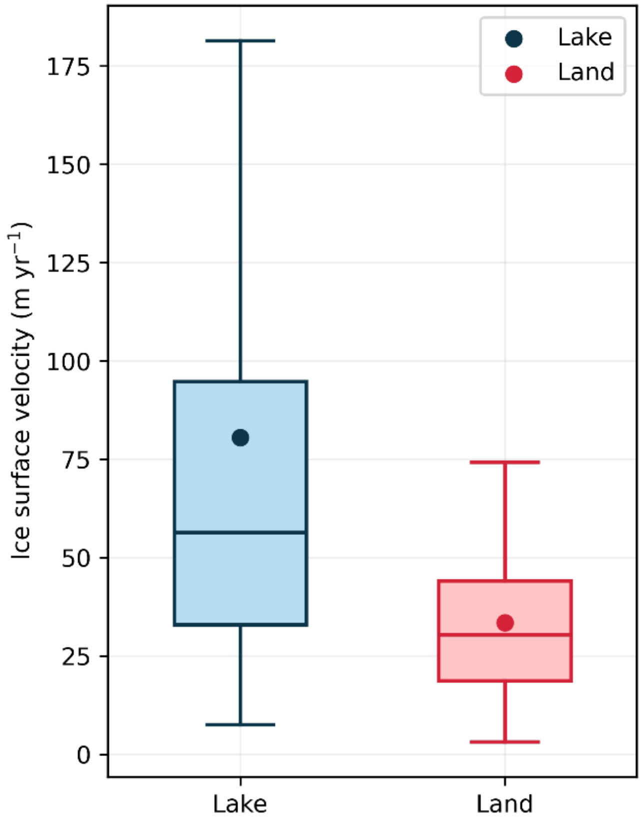

Supplementary Figure S2. Median velocity at 500 m up-ice from the terminus during the year 2000, aggregated from all lake-terminating (blue) and land-terminating (red) glaciers (*n* = 86). Data extracted from the ITS_LIVE V2 mosaic of annually averaged velocity during the year 2000, using an average of 13 pixels. Lake presence verified using the MEaSUREs GIMP 2000 Image Mosaic, Version 1 (Howat et al., 2017, updated 2018).

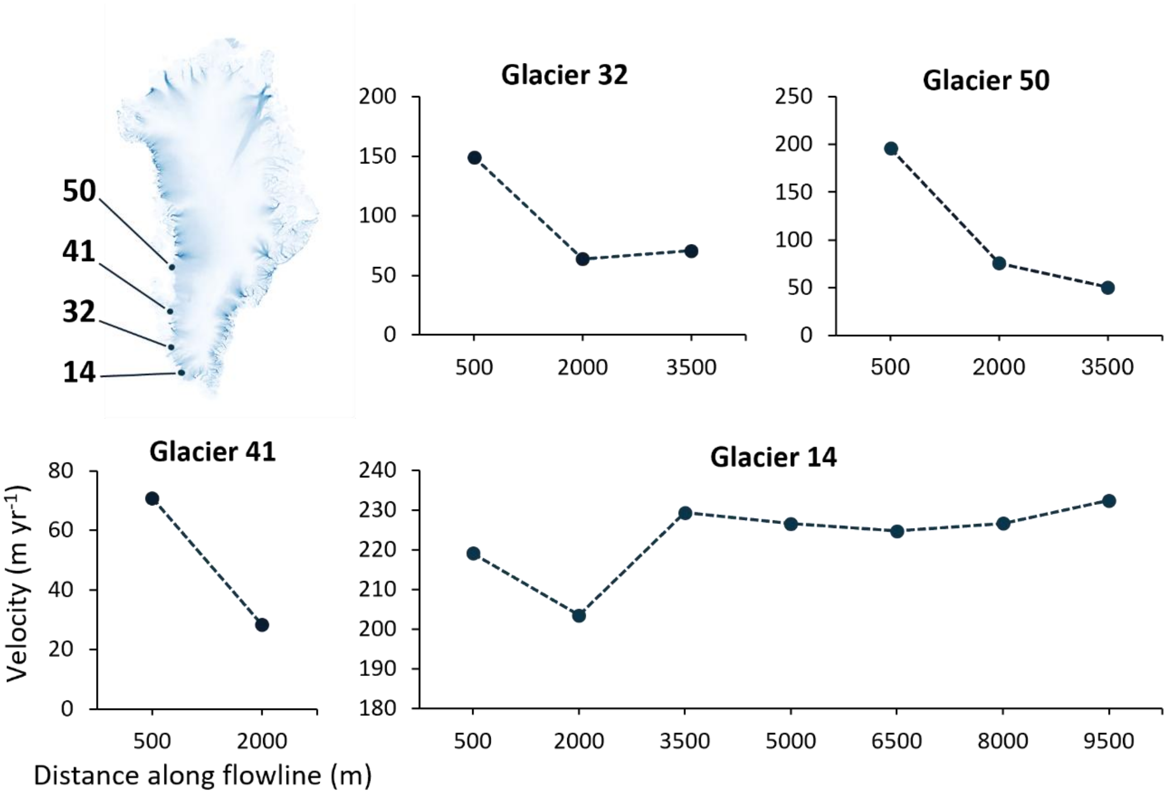

Supplementary Figure S3. Box-sampled velocity (m yr^{-1}) at four sample glaciers which show particularly extreme behaviour, including especially significant increases in velocity between 2000 m and 500 m up-ice at glaciers 32, 41 and 50. Note variable *y* axis ranges.

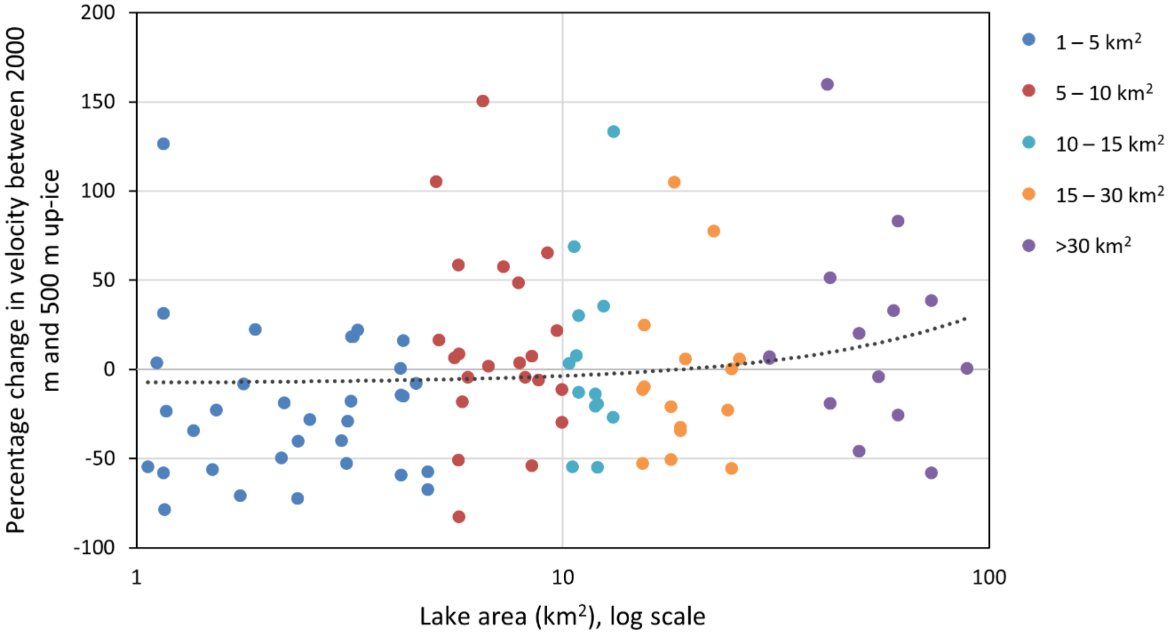

Supplementary Figure S4. Percentage change in velocity between 2000 m and 500 m up-ice at each lake-terminating glacier, scattered against lake area. Marker colours reflect lake area bin.

Lake area bin	U-statistic	*p*-value
1-5 km²	**114.0**	**0.015**
5.01-10 km²	139.0	0.905
10.01-15 km²	71.0	0.503
15.01-30 km²	54.0	0.200

Supplementary Table S2. Statistical comparison of differences between the percentage change in velocity from 2000 m to 500 m up-ice at glaciers flowing into lakes > 30 km^2 and glaciers with lakes within each other lake area bin, using a Mann-Whitney U test for difference and a significance threshold of $p \leq 0.05$ (highlighted in bold).

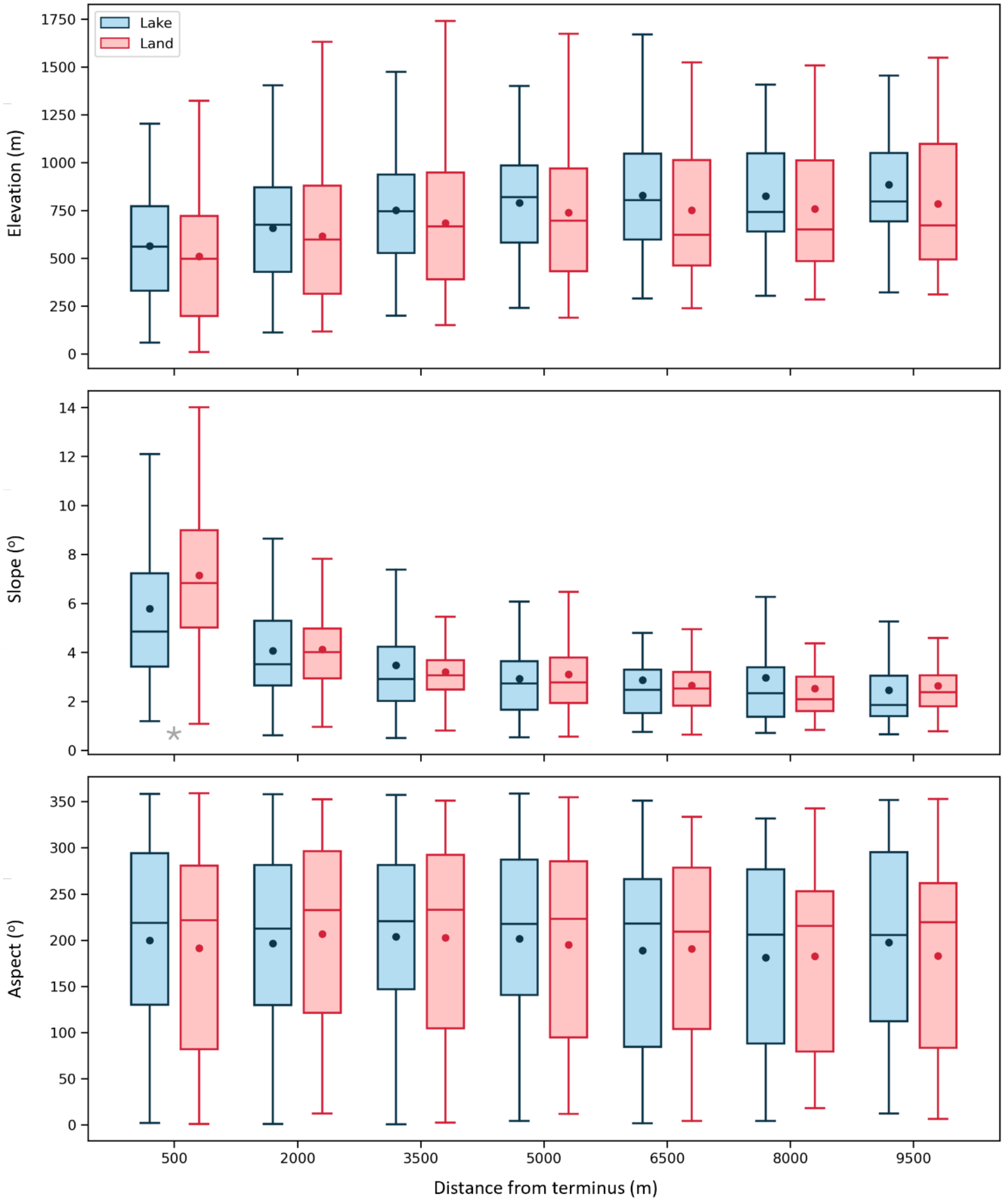

Supplementary Figure S5. Median box-sampled elevation, slope and aspect at each flowline location, aggregated from all lake-terminating (blue) and land-terminating (red) glaciers within each ice sheet region. The spread of velocity values across the sample population is shown by the box height. Asterisks mark box pairs with distributions which are statistically different. Sample numbers are displayed in Table S3.

Variable	Distance from terminus (m)	Lake sample number	Land sample number	U-statistic	*p*-value
**Elevation**	500	102	102	5942	0.0794
	2000	97	102	5535	0.1480
	3500	77	94	4178	0.0829
	5000	60	87	2957	0.1721
	6500	46	68	1823	0.1354
	8000	34	56	1110	0.1899
	9500	28	46	795	0.0935
**Slope**	500	102	102	3688.5	**0.0003**
	2000	97	102	4554	0.3338
	3500	77	94	3613	0.9864
	5000	60	87	2399	0.4068
	6500	46	68	1544.5	0.9126
	8000	34	56	984	0.7932
	9500	28	46	543	0.2627
**Aspect**	500	102	102	5402	0.6361
	2000	97	102	4615	0.4143
	3500	77	94	3577	0.8975
	5000	60	87	2647	0.8856
	6500	46	68	1542	0.9012
	8000	34	56	954	0.9900
	9500	28	46	705	0.5001

Supplementary Table S3. Statistical comparion of differences in elevation, slope and aspect at each flowline location between lake-terminating glaciers and land-terminating glaciers, using a Mann-Whitney U test for difference and a significance threshold of $p \leq 0.05$ (highlighted in bold).

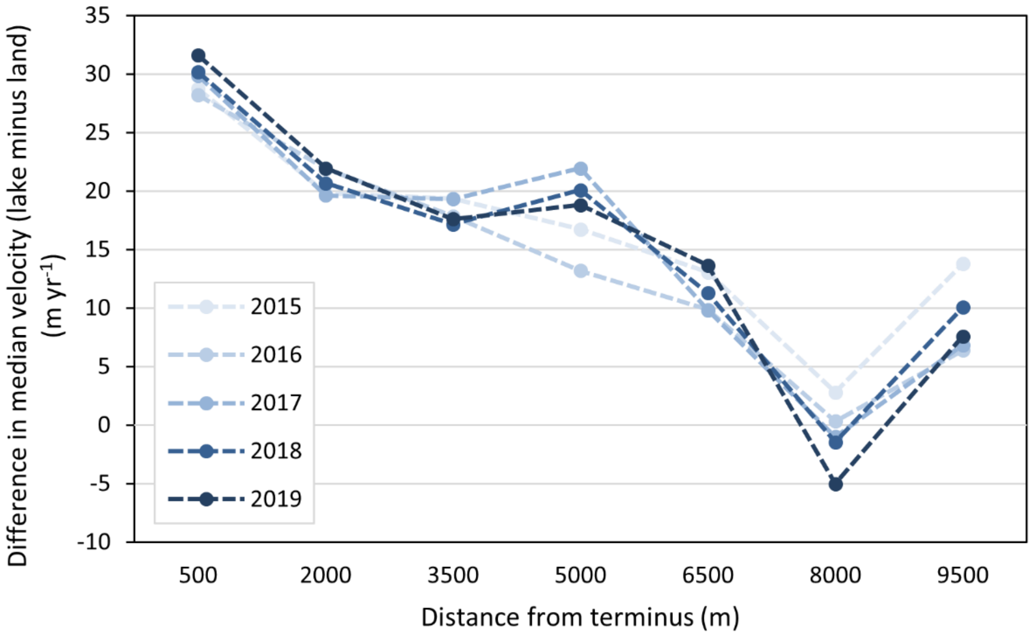

Supplementary Figure S6. Difference between the median velocity of all lake-terminating glaciers and all land-terminating glaciers at each flowline sampling location, years 2015 – 2019.

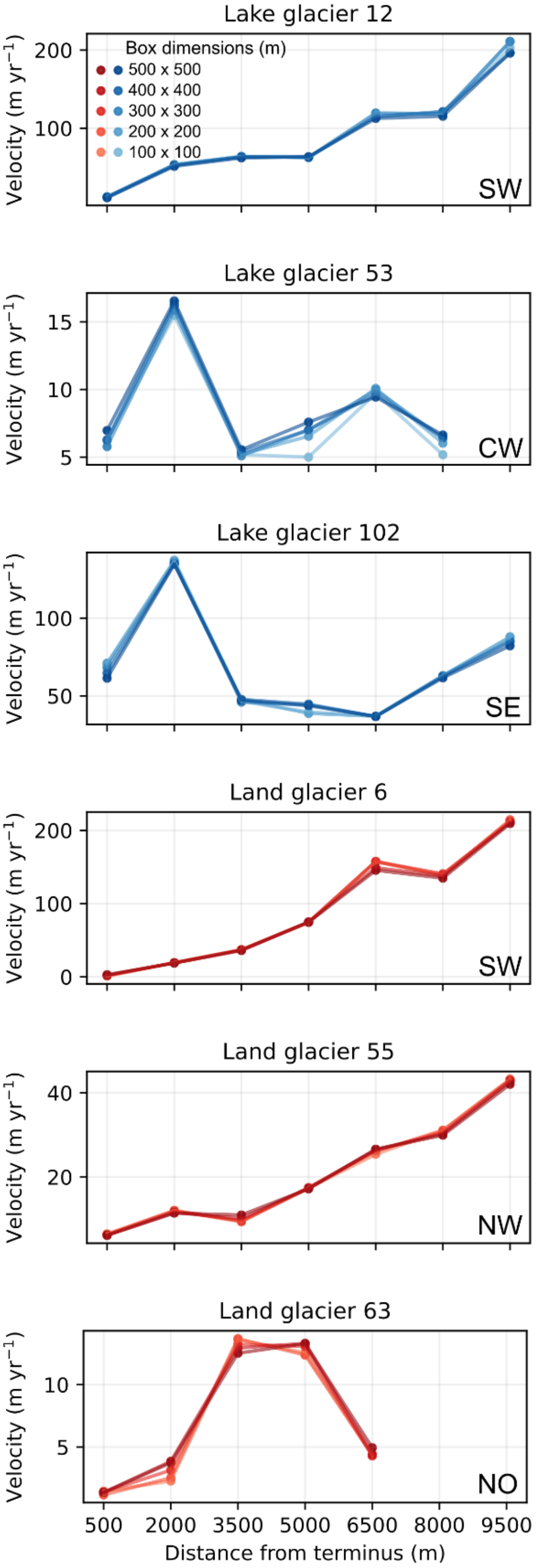

Distance	v range	v std. dev.	Mean % diff.
500	1.30	0.55	5.52
2000	2.36	0.94	2.12
3500	2.08	0.82	1.54
5000	1.25	0.54	1.08
6500	7.18	3.03	3.18
8000	6.00	2.30	2.38
9500	14.70	7.36	4.33

500	1.18	0.42	7.65
2000	1.07	0.42	3.23
3500	0.45	0.18	4.04
5000	2.59	0.98	17.90
6500	0.64	0.25	3.21
8000	1.46	0.58	11.19

500	9.72	4.08	7.58
2000	2.45	1.06	0.96
3500	1.71	0.77	2.01
5000	6.01	2.76	7.84
6500	0.34	0.14	0.47
8000	1.44	0.61	1.12
9500	5.85	2.37	3.41

500	1.77	0.72	47.05
2000	0.65	0.29	1.89
3500	1.10	0.44	1.40
5000	0.56	0.22	0.36
6500	12.27	5.71	4.34
8000	5.95	2.22	1.96
9500	5.14	2.07	1.23

500	0.37	0.16	3.08
2000	0.63	0.28	2.97
3500	1.63	0.66	7.75
5000	0.33	0.12	0.85
6500	1.19	0.48	2.18
8000	1.20	0.51	2.07
9500	1.21	0.49	1.36

500	0.30	0.11	9.77
2000	1.58	0.70	28.49
3500	1.17	0.48	4.56
5000	0.94	0.43	4.08
6500	0.65	0.27	6.05

Supplementary Figure S7. Plots display the median velocity value extracted within boxes of a range of dimensions (100 x 100 m, 200 x 200 m, 300 x 300 m, 400 x 400 m and 500 x 500 m) at six glaciers spanning a range of regions and velocity regimes. Glacier region is denoted in the lower right corner of each plot. Tables show the range, standard deviation and mean percentage difference between velocities extracted from each box size at each flowline location.

Supplementary References

Ely, J.C., Clark, C.D., Ng, F.S.L. and Spagnolo, M. (2017) 'Insights on the formation of longitudinal surface structures on ice sheets from analysis of their spacing, spatial distribution, and relationship to ice thickness and flow', *Journal of Geophysical Research: Earth Surface*, 122(4), 961–972. https://doi.org/10.1002/2016JF004071.

GLIMS and NSIDC (2005, updated 2018): Global Land Ice Measurements from Space glacier database. Compiled and made available by the international GLIMS community and the National Snow and Ice Data Center, Boulder CO, U.S.A. http://doi.org/10.7265/N5V98602.

Howat, I. 2017, updated 2018. MEaSUREs Greenland Ice Mapping Project (GIMP) 2000 Image Mosaic, Version 1. Boulder, Colorado USA. NASA National Snow and Ice Data Center Distributed Active Archive Center. https://doi.org/10.5067/4RNTRRE4JCYD. [Accessed 3/11/25].

RGI Consortium. (2023). *Randolph Glacier Inventory - A Dataset of Global Glacier Outlines*. (NSIDC-0770, Version 7). Boulder, Colorado USA. National Snow and Ice Data Center. https://doi.org/10.5067/F6JMOVY5NAVZ.
